# Melatonin Treatment to Pomegranate Trees Enhances Fruit Bioactive Compounds and Quality Traits at Harvest and during Postharvest Storage

**DOI:** 10.3390/antiox10060820

**Published:** 2021-05-21

**Authors:** José M. Lorente-Mento, Fabián Guillén, Salvador Castillo, Domingo Martínez-Romero, Juan M. Valverde, Daniel Valero, María Serrano

**Affiliations:** 1 Department of Applied Biology, EPSO, University Miguel Hernández. Ctra. Beniel km. 3.2, 03312 Orihuela, Alicante, Spain; jlorente@umh.es; 2 Department of Food Technology, EPSO, University Miguel Hernández. Ctra. Beniel km. 3.2, 03312 Orihuela, Alicante, Spain; fabian.guillen@umh.es (F.G.); scastillo@umh.es (S.C.); dmromero@umh.es (D.M.-R.); jm.valverde@umh.es (J.M.V.); daniel.valero@umh.es (D.V.)

**Keywords:** *Punica granatum* L., antioxidants, anthocyanins, phenolics, firmness, colour

## Abstract

The effect of melatonin pomegranate tree treatments on fruit quality and bioactive compounds with antioxidant activity at harvest and during storage at 10 °C for 60 days was assayed in two consecutive years, 2019 and 2020. In the first year, trees were treated with 0.1, 0.3 and 0.5 mM of melatonin along the developmental fruit growth cycle, and results showed that bioactive compounds (total phenolics and total and individual anthocyanins) and antioxidant activity at harvest were higher in fruits from melatonin-treated trees than in controls. Other fruit quality parameters, such as firmness, total soluble solids and aril red colour, were also increased as a consequence of melatonin treatment. In fruit from control tress, firmness and acidity levels decreased during storage, while increases occurred on total soluble solids, leading to fruit quality reductions. These changes were delayed, and even maintenance of total acidity was observed, in fruit from melatonin-treated trees with respect to controls, resulting in a fruit shelf-life increase. Moreover, concentration of phenolics and anthocyanins and antioxidant activity were maintained at higher levels in treated than in control fruits during the whole storage period. In general, all the mentioned effects were found at the highest level with the 0.1 mM melatonin dose, and then it was selected for repeating the experiment in the second year and results of the first year were confirmed. Thus, 0.1 mM melatonin treatment could be a useful tool to enhance aril content on bioactive compounds with antioxidant activity and health beneficial effects and to improve quality traits of pomegranate fruit, at harvest and during postharvest storage.

## 1. Introduction

Pomegranate is an ancient edible fruit, originated in the Middle East area nowadays occupied by Iran and Afghanistan, and from this area, it was spread to other countries. Spain is the major producer and exporting country of pomegranate fruit in the European Union, with a cultivated area of 5716 ha and a production of 75,673 t in 2018 [1]. Arils are the edible portion of pomegranate fruit, which consist of around 70–80% juice and 30–20% seed, depending on cultivar. Aril colour, sugar and acid content and the presence of small and soft seeds, together with fruit size and external appearance, are the main attributes responsible for fruit quality [2,3]. In addition, pomegranate was considered, from ancient times, to have medicinal properties to treat heart pain, leucorrhoea, sterility, lung diseases, febrifuge or cholera, as reported by Hippocrates in the 4th century B.C. [3]. These and other health beneficial effects against several diseases, such as atherosclerosis, inflammatory and infective-mediated diseases, Alzheimer, diabetes, infarct brain ischemia and several types of cancer, have been scientifically proven recently and attributed to its content of anthocyanins and other phenolic compounds [4,5,6,7,8,9,10]. Anthocyanins are the pigments responsible for skin and aril colour and in both tissues, increases occur during maturation [11]. The major anthocyanins in arils are cyanidin 3-glucoside (cyn 3-gluc) and delphinidin 3-glucoseide (delp 3-gluc), followed by delphinidin 3,5-diglucoside (delp 3,5-di-gluc), cyaniding 3,5-di-glucoside (cyn 3,5-di-gluc) or pelargonidin 3-glucoside (pelg 3-gluc), depending on cultivar [11,12,13]. On the contrary, aril total phenolic concentration decreases abruptly in the initial stages of fruit development, and thereafter, the decrease is very small [11,12]. However, phenolic concentration in mature fruit, as well as anthocyanin concentration, is different depending on cultivar [11,12,14,15,16].

‘Mollar de Elche’ is the most cultivated pomegranate cultivar in Spain, which is very appreciated by consumers since its arils have high concentration of sugars, low acidity and small and soft seeds that can be easily eaten [17,18]. However, the anthocyanin content in skin and flesh of ‘Mollar de Elche’ cultivar is not high as compared with other worldwide-known cultivars, such as ‘Wonderful’, and its arils are slightly red-coloured and the skin has a cream-pink colour [2,19,20]. This low colouration of skin and arils makes it difficult for this cultivar to reach international markets. Research has been performed in the last years, aimed to solve this problem. Thus, water restrictions applied in summer, during the linear phase of fruit growth, have been reported to increase aril anthocyanin content [21]. Similar effects have been reported by treatments with methyl jasmonate [13] or salicylates [16] during on-tree pomegranate fruit development. On the other hand, pomegranate exhibits important quality losses during postharvest storage. The most important changes are fruit water and firmness losses and reduction in aril acidity, which lead to reduction of fruit acceptability in terms of freshness, juiciness and taste [2,3].

Melatonin was identified in 1995 in angiosperms plant species, and nowadays, it is considered as a plant growth regulator with effects in a wide range of plant physiological processes, including alleviation of the oxidative damages caused by different biotic and abiotic stresses and root and seed development [22,23]. In addition, recent reports have shown a role of melatonin on fruit ripening, although most of them have been focused on postharvest treatments [24,25]. Thus, 0.5 mM melatonin dipping treatment for 1 h delayed the ripening process in ‘Guifei’ mangoes throughout inhibition of ethylene and ABA biosynthesis [26]. Accordingly, melatonin postharvest treatment inhibited ethylene production and delayed ripening in banana fruit [27], which were dose-dependent in the range of 0.05 to 0.5 mM. Similar results have been reported in peaches and nectarines [28,29]. In tomato fruit, postharvest treatment with melatonin at 50 µM increased anthocyanin content. This effect was positively correlated to fruit ripening but negatively related to fruit senescence, since the proteins related with senescence were downregulated while anti-senescent proteins, such as catalase and peroxidase, increased in treated fruit [30]. However, postharvest fruit treatments have legal restrictions and consumers’ concerns. Thus, research about preharvest treatments with effect on fruit quality properties at harvest and during storage is needed.

In this sense, the effect of melatonin applied as preharvest treatment on fruit ripening process and quality traits has been evaluated in very few papers, showing different effects depending on fruit species, concentration, or application time. Thus, 0.1 and 0.01 mM of melatonin applied at pit-hardening inhibited ripening in sweet cherry fruits [31]. On the contrary, irrigation of tomato plant with 0.1 mM of melatonin increased sugar content and lycopene concentration in fruits, showing a positive effect on fruit ripening [32]. In apricot, foliar spray melatonin treatment increased yield and fruit weight, although no effect on on-tree ripening was observed [33]. In addition, increased apricot quality parameters at harvest and maintenance during storage have been found as a consequence of melatonin tree treatment during fruit development [34]. However, to the best of our knowledge, no literature is available regarding the effect of preharvest melatonin treatment of pomegranate trees on fruit quality properties at harvest and during storage, which has been the main objective of this paper, with special interest in bioactive compounds, such as anthocyanins and phenolics, and antioxidant activity.

## 2. Materials and Methods

### 2.1. Plant Material and Experimental Design

Pomegranate (*Punica granatum* L. cv. Mollar de Elche) trees, 12 and 13 years-old, were used for 2019 and 2020 experiments respectively, grown in the same commercial plot located at Elche (Alicante, Spain) and under similar agronomic practices for both years. Climatic conditions in the crop field (UTMX: 694,006.000 UTMY: 4,234,860.000) were: a semi-arid Mediterranean climate, with mean annual temperatures of 18.90 and 19.52 °C for 2019 and 2020, respectively; maximum temperatures in summer, from June to September, of 30.35 and 32.23 for 2019 and 2020, respectively; an accumulated rainfall of 285.43 and 233.37 mm for 2019 and 2020, respectively. In 2019, 3 blocks of 3 trees were selected for each treatment: control (distilled water) and melatonin (purchased from Sigma, Sigma-Aldrich, Madrid) at 0.1, 0.3 and 0.5 mM. These concentrations were chosen according to previous reports in which the effect of melatonin, applied as preharvest treatment, on quality properties of other fruit species was assayed [31,32,33,34]. Two litres per tree of freshly prepared solutions (containing 0.1% Tween 20) were applied to pomegranate trees with a mechanical mist sprayer, which was enough to wet the entire tree canopy. Five treatments were applied during the fruit growth cycle (30, 60, 90, 105 and 120 days after full blossom: T1, T2, T3, T4 and T5), with the last treatment being applied 3 days before harvesting. Fruit were harvested at commercial maturity stage, based on the characteristic fruit size, skin colour and soluble solids content (14–15 °Brix) of this cultivar. From each treatment and replicate, 15 pomegranate fruit, homogeneous in size and colour and without visual defects, were selected, grouped at random in lots of 5 fruits and transported to the laboratory in 2 h for storage at 10 °C and 80% RH. After 0, 30 and 60 days, one lot of each treatment and replicate was taken at random and used for the following analytical measures: firmness of the whole fruit, skin colour and colour, total soluble solids (TSS), titratable acidity (TA), total phenolics and anthocyanins, individual anthocyanins and hydrophilic total antioxidant activity (H-TAA) in the arils. In 2020, the 0.1 mM concentration was chosen, since it gave the best results in terms of fruit quality parameters and content on bioactive compounds. Treatments were performed on 3 replicates of 3 trees which were different from those used in the 2019 experiment but grown in the same commercial plot. Treatments and storage conditions were similar to 2019, although samples during storage were taken at shorter intervals, after 0, 15, 30, 45 and 60 days of storage. Thus, from each treatment and replicate, 25 pomegranate fruit were selected, grouped in lots of five fruit, and one lot from each replicate and treatment was taken for each sampling date during storage for analytical measures.

### 2.2. Fruit Quality Parameters

Fruit firmness was determined in each individual fruit of each replicate by using a TX-XT2i Texture Analyser (Stable Microsystems, Godalming, UK) interfaced to a personal computer. A flat steel plate was mounted on the machine which measured the fruit equatorial diameter and applied a force that achieved a 5% deformation of the fruit diameter. Firmness results were expressed as the force-deformation ratio (N mm^−1^). Skin colour was measured on three points of the equatorial fruit perimeter by using a Minolta colorimeter (CRC200, Minolta Camera Co., Kantō, Tokio, Japan) and colour was expressed as Hue (arctg b*/a*) according the CIELab coordinates. Pomegranate fruit were cut in two halves, and again, three colour readings were taken in the arils of each fruit to measure internal colour as Hue. Arils from the five fruit of each replicate and treatment were taken and combined to obtain a homogeneous sample in which the following parameters were measured. About 50 g of arils were squeezed through a cotton cloth and the juice used to quantify TSS and TA in duplicate. TSS was determined by using a digital refractometer, Atago PR-101 (Atago Co. Ltd., Tokyo, Japan), at 20 °C, and expressed as g kg^−1^. One mL of the juice was diluted in 25 mL of distilled H_2_O and used to measure titratable acidity (TA) by automatic titration (785 DMP Titrino, Metrohm) with 0.1 N NaOH up to pH 8.1, and results were expressed as g kg^−1^ malic acid equivalent.

### 2.3. Total Phenolics and Total and Individual Anthocyanins Quantification

To perform phenolic extraction, 5 g of arils from each replicate were homogenised with 10 mL of water:methanol (2:8) containing 2 mM NaF (to inactivate polyphenol oxidase activity and prevent phenolic degradation) for 30 s in a homogeniser (Ultraturrax, T18 basic, IKA, Berlin, Germany). The extracts were centrifuged at 10,000× *g* for 10 min at 4 °C and the supernatant was used to quantify, in duplicate, total phenolic content using the Folin–Ciocalteu reagent, as described by García-Pastor et al. [13]. Results were expressed as mg gallic acid equivalent kg^−1^. Anthocyanins were extracted by homogenising 5 g of arils from each replicate with 15 mL of methanol/formic acid/water (25:1:24, *v*/*v*/*v*) and then, the extracts were centrifuged at 10,000× *g* for 10 min at 4 °C. Total anthocyanin concentration was determined, in duplicate, in the supernatant by reading absorbance at 520 nm, as previously reported [13], and results were expressed as mg kg^-1^ of cyanidin 3-glucoside equivalent (cyn 3-glu, molar absorption coefficient of 26,900 L cm^−1^ mol^−1^ and molecular weight of 449.2 g mol^−1^). For individual anthocyanin quantification, these extracts were filtered through a 0.45 μm PVDF filter (Millex HV13, Millipore, Bedford, MA, USA), and then, two samples of 20 µL of each extract were injected into an HPLC system (Agilent HPLC 1200 Infinity series) working with the chromatographic conditions previously reported [16]. Chromatograms were recorded at 520 nm and quantification was performed by using calibration curves carried out with cyanidin 3-*O*-glucoside (cyn 3-gluc), cyanidin 3,5-*O*-di-glucoside (cyn 3,5-*O*-di-gluc), pelargonidin 3-*O*-glucoside (plg 3-gluc) and delphinidin 3-*O*-glucoside (dlp 3-gluc), purchased from Sigma-Aldrich (Darmstadt, Germany). Results were expressed as mg kg^−1^.

Hydrophilic total antioxidant activity (H-TAA) was quantified according to Serrano et al. [35]. Briefly, 5 g of arils from each sample were homogenised in 10 mL of 50 mM phosphate buffer, pH = 7.8, and centrifuged at 15,000× *g* for 15 min at 4 °C. The supernatant was used for H-TAA determination by using the enzymatic system composed of 2,20-azino-bis-(3-ethylbenzothiazoline-6-sulfonic acid) diammonium salt (ABTS), horse radish peroxidase enzyme (HRP) and hydrogen peroxide as its oxidant substrate, in which ABTS^+^ radicals are generated and monitored at 730 nm. The decrease in absorbance after 60 s of adding the pomegranate extract was calculated and used to quantify H-TAA by comparison with a calibration curve performed with trolox ((R)-(+)-6-hydroxy- 2,5,7,8-tetramethyl-croman-2-carboxylic acid) (0–20 nmol) from Sigma (Madrid, Spain), and results are expressed as mg kg^−1^ trolox equivalent (TE).

### 2.4. Statistical Analyses

For both experimental years, a factorial design with melatonin treatments and storage time with triplicate samples (*n* = 3) was performed. Analysis of variance (ANOVA) was performed by using the SPSS software version 20 (SPSS Inc., Chicago, IL, USA) and means were compared by Tukey’s test. Differences at *p* < 0.05 were considered significant. Data are represented as means ± standard error (SE).

## 3. Results

### 3.1. Effect of Preharvest Melatonin Treatment on Bioactive Compounds and Antioxidant Activity

**Figure 1:**
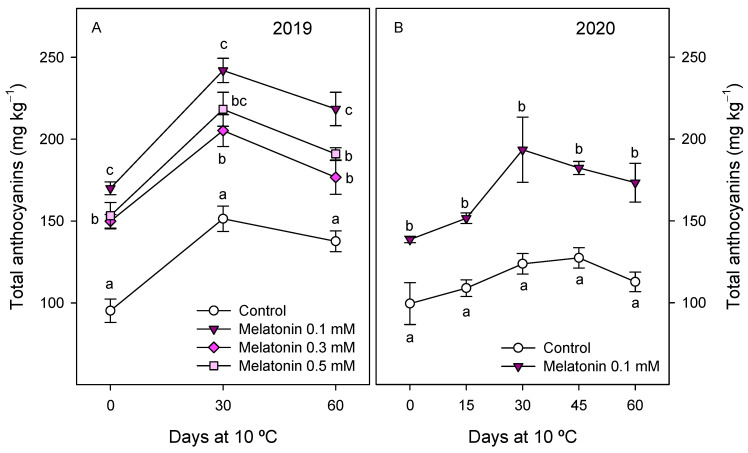
Total anthocyanin concentration in arils of pomegranate fruit from control and melatonin-treated trees during storage at 10 °C in 2019 (**A**) and 2020 (**B**) experiments. Data are the mean ± SE. Different letters (a–c) show significant differences (*p* < 0.05) among treatments for each sampling date.

**Figure 2:**
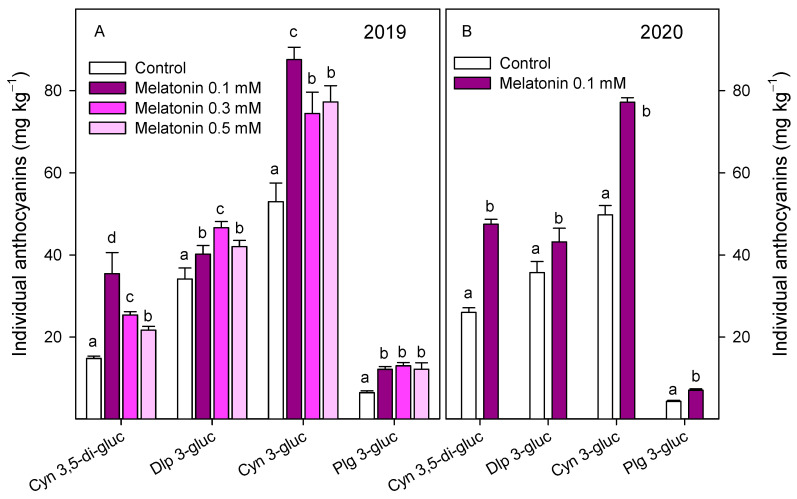
Individual anthocyanin concentration in arils of pomegranate fruit from control and melatonin-treated trees at harvest in 2019 (**A**) and 2020 (**B**) experiments. Data are the mean ± SE. Different letters (a–c) show significant differences (*p* < 0.05) among treatments for each individual anthocyanin.

Anthocyanin concentration in fruit from control trees at harvest, in the 2019 experiment, was 95.2 ± 7.1 mg kg^−1^, and significantly higher (*p* < 0.05) in the arils of pomegranates from melatonin-treated trees, at 150–170 mg kg^−1^. An increase in total anthocyanin content was found in arils of all pomegranate fruit during the first 30 days of storage and a decreasing trend was observed thereafter. However, total anthocyanin concentration was maintained at higher levels in arils of pomegranates from treated trees during the whole storage period, with the highest concentration being found for 0.1 mM melatonin treatment (Figure 1A). Similar trends in total anthocyanin concentration were observed in the 2020 experiment, although significantly higher (*p* < 0.05) values were found in arils of pomegranate from melatonin-treated trees than in controls, at harvest and during storage (Figure 1B). Four individual anthocyanins were identified and quantified, at harvest, in pomegranate arils in 2019 and 2020 experiments, and similar profile was found in fruits from control and treated trees, the major anthocyanin being cyn 3-gluc, followed by dlp 3-gluc, cyn 3,5-di-gluc and plg 3-gluc (Figure 2). Concentrations of these anthocyanins were significantly (*p* < 0.05) enhanced by preharvest melatonin treatments, and in general, the major effect was observed for the 0.1 mM dose in the 2019 experiment, with 1.7-, 1.2-, 2.4- and 1.9-fold increases for cyn 3-gluc, dlp 3-gluc, cyn 3,5-di-gluc and plg 3-gluc, respectively (Figure 2A). However, for dlp 3-gluc, the highest increase was recorded for the 0.3 mM dose. These increases on individual anthocyanin concentrations at harvest as a consequence of preharvest melatonin treatments were confirmed in the 2020 experiment, in which a 0.1 mM dose was applied (Figure 2B).

**Figure 3:**
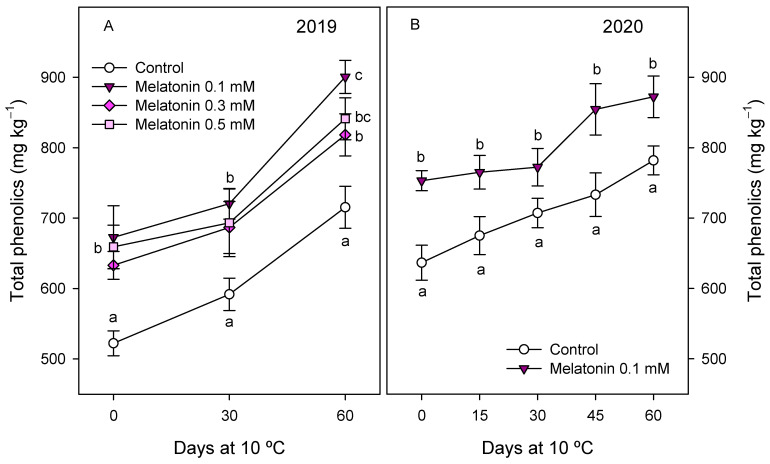
Total phenolic concentration in arils of pomegranate fruit from control and melatonin-treated trees during storage at 10 °C in 2019 (**A**) and 2020 (**B**) experiments. Data are the mean ± SE. Different letters (a–c) show significant differences (*p* < 0.05) among treatments for each sampling date.

Total phenolic concentration at harvest in the 2019 experiment was 522.2 ± 17.8 mg kg^−1^ in arils of pomegranate from control trees and significantly higher (*p* < 0.05), ca. 650 mg kg^−1^, in those from melatonin-treated trees, without significant differences among doses. During storage, aril phenolic concentration increased irrespective of treatment, although significantly higher levels were maintained in fruit from melatonin-treated trees with respect to controls (Figure 3A). Similar effects of melatonin preharvest treatments on both phenolic concentration and behaviour during storage were found in the 2020 experiment when the 0.1 mM dose was used (Figure 3B).

**Figure 4:**
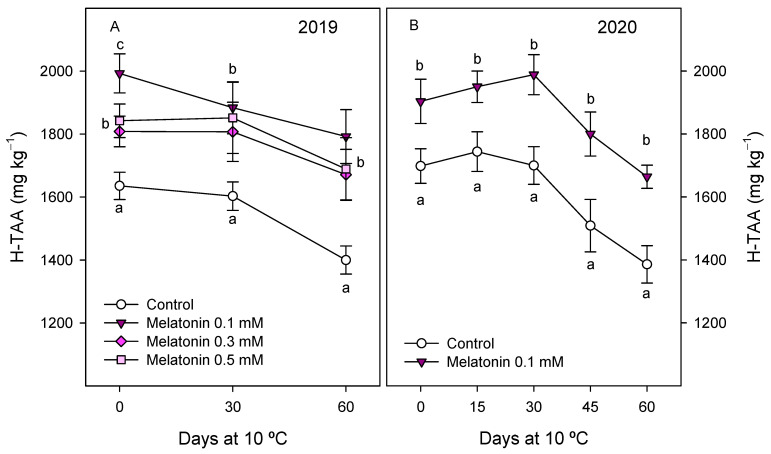
Hydrophilic total antioxidant activity (H-TAA) in arils of pomegranate fruit from control and melatonin-treated trees during storage at 10 °C in 2019 (**A**) and 2020 (**B**) experiments. Data are the mean ± SE. Different letters (a–c) show significant differences (*p* < 0.05) among treatments for each sampling date.

H-TAA at harvest was significantly higher (*p* < 0.05) in arils of pomegranate from melatonin-treated trees than in controls, with these increases being ca. 20% for the 0.1 mM dose and ca. 10–12% for the 0.3 and 0.5 mM doses in the 2019 experiment (Figure 4A). Enhanced H-TAA at harvest was also obtained in the 2020 experiment as a consequence of 0.1 mM melatonin treatment (Figure 4B). These values of H-TAA were maintained without significant changes during the first 30 days of storage in arils of pomegranates from control and treated tress, and decreased thereafter, in the 2019 and 2020 experiments (Figure 4). However, it is worth mentioning that higher levels of H-TAA were found for all melatonin treatments in the 2019 experiment (ca. 20% for 0.1 mM melatonin treatment) with respect to controls during the whole storage period (Figure 4A). H-TAA was also enhanced at harvest and maintained at highest levels during storage in fruits from 0.1 mM melatonin-treated trees with respect to controls in the 2020 experiment (Figure 4B).

### 3.2. Effect of Melatonin Treatment on Quality Parameters during Storage

**Figure 5:**
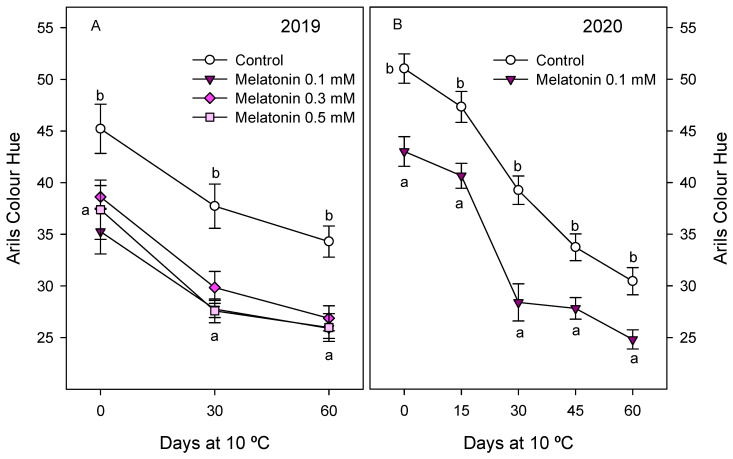
Colour Hue in the arils of pomegranate fruit from control and melatonin-treated trees during storage at 10 °C in 2019 (**A**) and 2020 (**B**) experiments. Data are the mean ± SE. Different letters (a,b) show significant differences (*p* < 0.05) among treatments for each sampling date.

**Figure 6:**
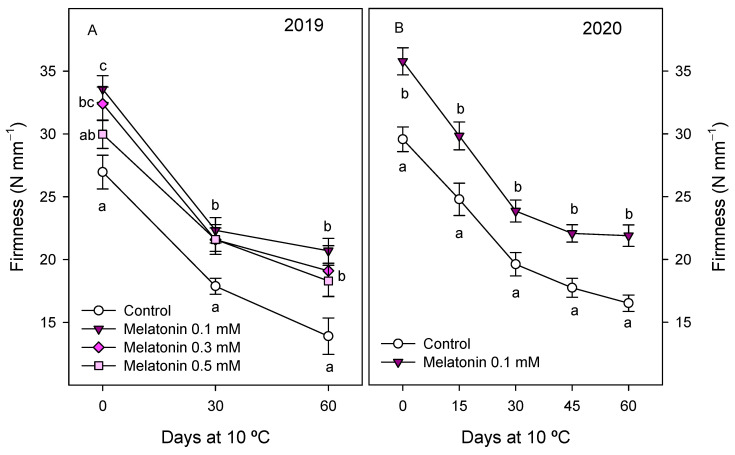
Firmness of pomegranate fruit from control and melatonin-treated trees during storage at 10 °C in 2019 (**A**) and 2020 (**B**) experiments. Data are the mean ± SE. Different letters (a–c) show significant differences (*p* < 0.05) among treatments for each sampling date.

Quality parameters of pomegranate fruit, such as external and internal colour, firmness, TSS and TA, were also affected by melatonin treatments. Thus, skin colour at harvest, expressed as Hue angle, was significantly lower (*p* < 0.05) in fruit from 0.1 mM melatonin-treated trees than in controls in both years, while no significant effect was observed for the 0.3 and 0.5 mM doses (Supplementary Figure S1). However, aril colour was affected by all the melatonin assayed doses, with values of Hue angle at harvest of 45.21 ± 2.38 in fruit from control trees and significantly lower (*p* < 0.05), from 35 to 38, in those from treated trees, without significant differences among melatonin-applied doses (Figure 5A). Lower values of Hue angle in both skin and arils, as a consequence of melatonin treatments, mean a more intense red colour. These results were also observed in the 2020 experiment, that is, significantly lower (*p* < 0.05) Hue values in skin and arils in fruit from treated trees than in controls (Supplementary Figure S1B and Figure 5B). Fruit firmness at harvest was significantly higher (*p* < 0.05), ca. 20%, in fruit from 0.1 and 0.3 mM melatonin-treated trees with respect to controls in 2019, and decreases were observed during storage, especially for the first 30 days, although it is worth noting that firmness values were maintained at higher levels in melatonin-treated fruit during the whole storage period (Figure 6A). Accordingly, in the 2020 experiment, significantly higher (*p* < 0.05) values of firmness were obtained in fruit from melatonin-treated trees than in controls, and these differences were evident during the whole storage period (Figure 6B).

TSS content was 145 and 148 in control fruit in 2019 and 2020 experiments respectively, and 5% higher in fruit from 0.1 mM melatonin-treated trees (in both years), while no significant effect was found in 0.3 and 0.5 mM treatments in 2019. During storage, increase trends in TSS were observed for control and treated fruit, although significantly higher (*p* < 0.05) levels in 0.1 mM melatonin-treated fruit than in controls were evident until the last sampling date (Supplementary Figure S2) for 2019 and 2020 experiments. On the contrary, similar values of TA at harvest were observed in fruit from control and treated trees, but a 20% decrease in TA level occurred in controls during storage, while this decrease was only 2% and 5% in fruit from 0.1 mM melatonin-treated trees for 2019 and 2020, respectively (Supplementary Figure S3).

## 4. Discussion

Pomegranate fruit have a wide range of bioactive compounds, the most important being phenolics, including anthocyanins, hydrolysable tannins and other complex flavonoids, and ascorbic acid, which are found at high concentrations as compared with other fruits of the Mediterranean diet, although different contents have been reported depending on cultivar, ripening stage, cultural practices and environmental conditions [12,15,19,36]. These bioactive compounds have antioxidant capacity and are responsible for the health beneficial effects attributed to pomegranate fruit consumption [4,6,7,8,10]. Results of the present experiments show that preharvest melatonin treatments enhanced total phenolic compound and total and individual anthocyanin concentrations at harvest, leading to increases in H-TAA, with these effects being maintained during storage. Thus, preharvest melatonin treatments would improve the pomegranate health beneficial effects for human consumption since phenolic compounds, including anthocyanins, are a wide range of secondary metabolites with preventive effects on a wide range of chronic and age-related diseases, such as hypertension, obesity, diabetes, cardiovascular, neurodegenerative and oncologic diseases [9,37,38,39]. No previous reports are available in the literature regarding the effect of melatonin treatment on pomegranate bioactive compounds, and little information exists on other fruit species. For instance, 0.1 mM melatonin treatment of tomato plants increased antioxidant compounds, such as lycopene, β-carotene, total phenolics and flavonoids, and antioxidant activity in tomato fruit when plants were grown under acid rain stress conditions [40]. Accordingly, leaves’ treatment of sweet cherry trees with 0.05 or 0.1 mM of melatonin improved total phenol, flavanol, anthocyanin and ascorbic concentrations in fruit at harvest [41]. However, the effects of melatonin postharvest treatments on increasing fruit bioactive compounds and antioxidant activity have been addressed in more fruit species. For instance, in ‘Santa Rosa’ plum, postharvest dipping treatment with 0.1 mM melatonin led to higher ascorbic acid, total phenolics and antioxidant activity levels during storage as compared with controls [42], as well as on strawberry treated with 0.1 or 1 mM of melatonin [43]. In addition, dipping treatment of nectarine fruit with 0.25, 0.5 and 1 mM of melatonin for 30 min and of peach fruit with 0.1 mM for 10 min minimised phenolic losses during storage at 1 °C [29,44]. Accordingly, 0.1 mM melatonin dipping treatment of sweet cherry increased anthocyanin content during storage due to an induced overexpression of two key genes coding for dihydroflavonol 4-reductase (DFR) and anthocyanidin 3-*O*-glucosyltransferase (UFGT), which are enzymes involved in the last steps of anthocyanin biosynthesis [45]. Moreover, specifically in ‘Malas Saveh’ pomegranate, Aghdam et al. [46] have reported recently that postharvest 0.1 mM melatonin treatment leads to higher content of anthocyanin and phenolics during storage at 4 °C due to higher activity of phenylalanine ammonia lyase (PAL), the first enzyme involved in the phenylpropanoid pathway activity, and increased intracellular NADPH supply.

‘Mollar de Elche’ pomegranate is very appreciated by consumers due to its high content of sugars and low acidity, which confer a sweet taste, being also very aromatic, although arils have a pale red colour as compared with other cultivars grown in Spain, such as ‘White’, ‘CG8′, ‘Katirbasi’ or ‘Wonderful’ [14,18], or other Tunisian, Turkish, Croatian or Iranian cultivars [2,12,15,19,20], depreciating its commercial value in international markets which are used to buy more red-coloured cultivars. Results of the present research would contribute to increase the profit of this crop since melatonin-treated fruit had a deeper aril red colour than controls (lower Hue angle) at harvest and during storage, as well as total anthocyanin concentration, showing a stimulation of the anthocyanin biosynthesis by melatonin treatment. Nevertheless, anthocyanin profile was not affected by preharvest melatonin treatment, irrespective of the applied concentration, with cyn 3-gluc being the major anthocyanin, followed by dlp 3-gluc, cyn 3,5-di-gluc and plg 3-gluc, according to previous reports regarding individual anthocyanin concentration of this cultivar [13,14,21]. Anthocyanin profile seems to be similar in all pomegranate cultivars, although their relative proportion of each one is different. Thus, cyn 3-gluc was also reported as the predominant anthocyanin in ‘Wonderful’ pomegranate [47], while dlp 3-gluc was the major anthocyanin for some Croatian cultivars [12], plg 3,5-di-gluc for Iranian cultivars [48] and cyn 3,5-di-gluc for Tunisian cultivars [49].

H-TAA was higher in arils of fruit from melatonin-treated trees than in controls, at harvest and during storage, the highest effect being observed for the 0.1 mM dose. Antioxidant activity in pomegranate arils, as well as in other fresh fruit species, is mainly due to hydrophilic compounds such as phenolics, including anthocyanins, and ascorbic acid [13,35,36,50]. In agreement with these previous reports, the present results show enhanced concentrations of total phenolics and total and individual anthocyanins at harvest in treated fruit, which could be responsible for the higher H-TAA found in treated than in control fruits. However, during storage, total phenolic concentration increased from 30 to 60 days of storage, while anthocyanin concentration increased from day 0 to day 30 and remained stable thereafter. Thus, the observed reductions in H-TAA from 30 to 60 days of storage should be attributed, apart from phenolics and anthocyanins, to ascorbic acid, an important hydrophilic antioxidant compound which decreases sharply during storage in ‘Mollar de Elche’ pomegranate fruit [13], as well as in ‘Wonderful’ [36] and ‘Malas Saveh’ [51] cultivars.

Fruit firmness, skin and aril colour and aril TSS and TA levels are the main attributes responsible for pomegranate fruit quality [2,3,17]. Then, the higher firmness and TSS and the lower skin and aril Hue angle values found at harvest in pomegranate fruit from melatonin-treated trees with respect to controls show that they had higher-quality attributes than controls. In addition, increases in TSS and decreases in fruit firmness and skin and arils Hue angle show the normal evolution of the ‘Mollar de Elche’ and other pomegranate cultivars’ postharvest ripening process [16,51,52], which were delayed by preharvest melatonin treatments. Moreover, TSS and TA are important quality parameters affecting sweetness and sourness respectively, and contributing to fruit taste. Then, the TSS increase and the reduction in TA in pomegranate arils during storage lead to increment the TSS/TA ratio, which is organoleptically appreciated as over-ripened fruit without the freshness, juiciness and taste required by consumers [2,3,11,16,50]. Thus, taking into account the evolution of all these quality parameters (mainly decreases in fruit firmness and TA in the arils), the maximum storage period at 10 °C of control pomegranate fruit with high quality for consumption in 2019 and 2020 experiments was 30 days, while this period was extended up to 60 days in fruit from all melatonin-treated trees. In fact, fruit firmness and arils TA in fruit from all melatonin-treated trees after 60 days of storage were even higher than in controls after 30 days of storage. In addition, as no significant differences were observed in these parameters among 0.1, 0.3 and 0.5 mM melatonin treatments, the application of the 0.1 mM dose could be enough for practical purposes. No previous reports are available regarding the effects of preharvest melatonin treatments on delaying fruit ripening and maintaining fruit quality attributes during storage for comparative purposes. However, postharvest dipping treatments have been reported to maintain fruit quality traits and extend shelf-life in several fruit species, such as banana, peach, pear, kiwi or strawberry, and even in pomegranate, as recently revised by Ze et al. [53], although the applied dose and time dipping was different for each fruit species. These effects were attributed to a reduced accumulation of reactive oxygen species (ROS) due to increases in the enzymatic activities and gene expressions of antioxidant enzymes. On the other hand, in climacteric fruit, such as ‘Colorado’ and ‘Mikado’ apricot, an effect of 0.1 mM melatonin treatments on delaying the postharvest ripening process at 1 and 8 °C has been observed recently, which was attributed to a reduced ethylene production in both cultivars and both storage temperatures [54]. Similar effects were observed by postharvest melatonin treatments on mangoes [26], banana [27], peaches [28], nectarines [29], apples [55] and pears [56], which were attributed to a reduced expression of genes codifying for ACC-synthase and ACC-oxidase. However, pomegranate is a non-climacteric fruit and ethylene production was very low in all fruit during storage, irrespective of treatments (data not shown). Accordingly, postharvest melatonin treatment also delayed the postharvest ripening process in non-climacteric fruits, such as sweet cherry [29], which was not mediated by reduction in ethylene production.

In addition, we recently found that 0.1 mM melatonin preharvest treatment increased crop yield of pomegranate trees [54], as well as in tomato plants under acid rain [40] or water-deficit stress conditions [57], with these effects being attributed to the ability of melatonin treatment on boosting the stress tolerance of tomato plants, throughout an increase in leaf chlorophyll content and photosynthetic rate. Thus, given the semi-arid climate conditions of Southern Spain, the melatonin treatment could have an additional effect, increasing tree net photosynthesis rate and productivity throughout enhancement of tree tolerance to heat and drought stresses [23,58], and in turn, leading to enhanced economic profit for growers.

## 5. Conclusions

This is the first report showing that melatonin applied as preharvest treatment has a significant effect on increasing pomegranate fruit quality parameters and their content in bioactive compounds with antioxidant properties at harvest and during postharvest storage at 10 °C, with the best results being found for the 0.1 mM dose. Overall, these results would permit to confirm that melatonin was effective on delaying the postharvest ripening process of pomegranate and extending fruit shelf-life. Thus, melatonin could be a reliable, feasible and cost-effective tool to be used in order to increase pomegranate fruit quality parameters, especially aril colour and antioxidant compounds, at harvest and to maintain them during storage.

## Data Availability

Data have been addressed in the manuscript and in the Supplementary Material.

## Supplementary Materials

The following are available online at https://www.mdpi.com/article/10.3390/antiox10060820/s1, Figure S1: Skin colour Hue of pomegranate fruit from control and melatonin-treated trees during storage at 10 °C, Figure S2: Total soluble solids (TSS) in arils of pomegranate fruit from control and melatonin-treated trees during storage at 10 °C, Figure S3: Titratable acidity (TA) in arils of pomegranate fruit from control and melatonin-treated trees during storage at 10 °C.
