# Supplementary material for: Melatonin Treatment to Pomegranate Trees Enhances Fruit Bioactive Compounds and Quality Traits at Harvest and during Postharvest Storage
Source: Antioxidants (Basel). 2021 May 21;10(6):820. doi: 10.3390/antiox10060820 (PMC8224012; doi:10.3390/antiox10060820)
Supplement: Supplementary file 1 [file antioxidants-10-00820-s001.zip › antioxidants-1206068-supplementary.pdf]

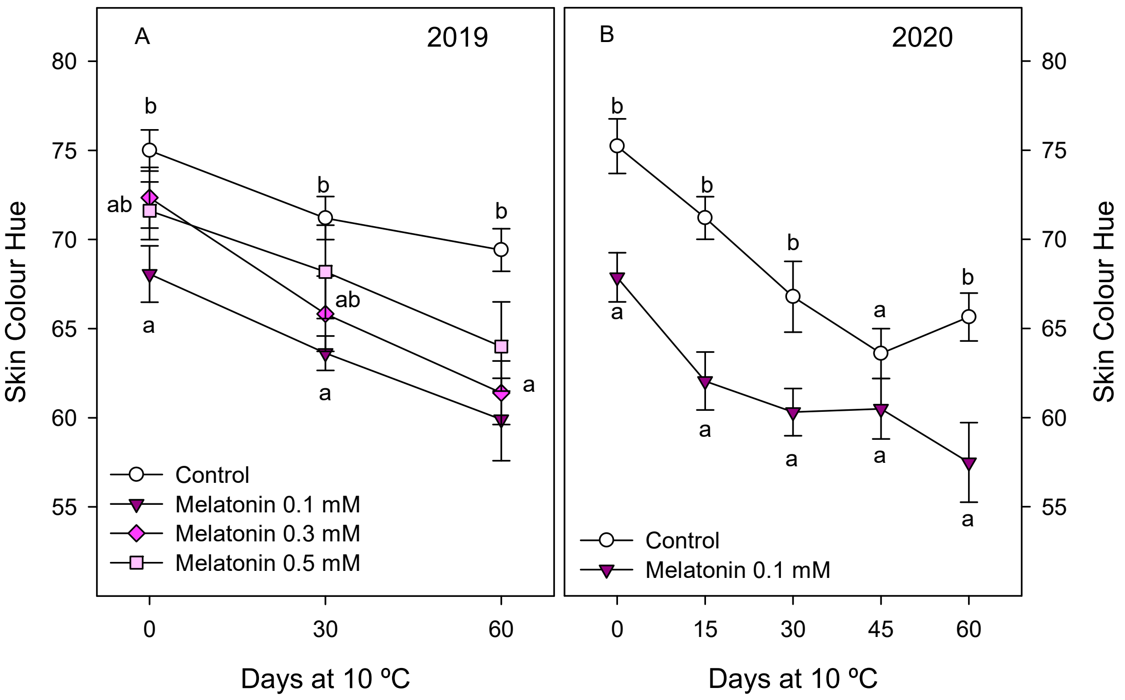

Figure S1. Skin colour hue of pomegranate fruit from control and melatonin-treated trees during storage at 10 °C. Data are the mean ± SE. Different letters show significant differences ($p < 0.05$) among treatments for each sampling date.

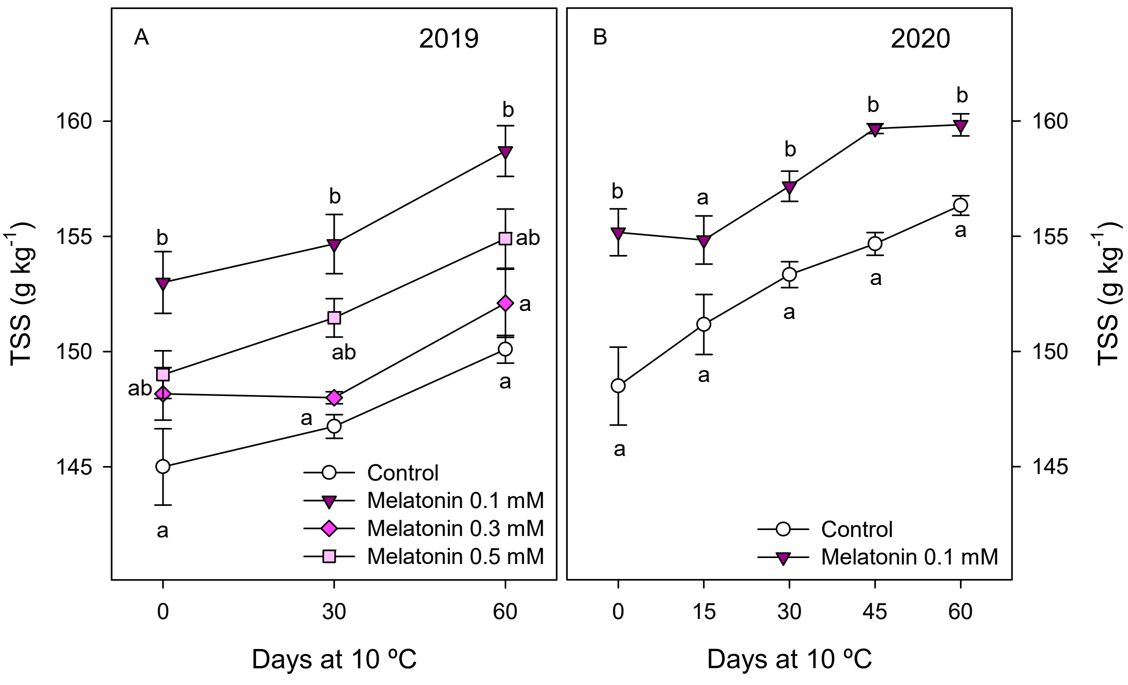

Figure S2. Total soluble solids (TSS) in arils of pomegranate fruit from control and melatonin-treated trees during storage at 10 °C. Data are the mean ± SE. Different letters show significant differences ($p < 0.05$) among treatments for each sampling date.

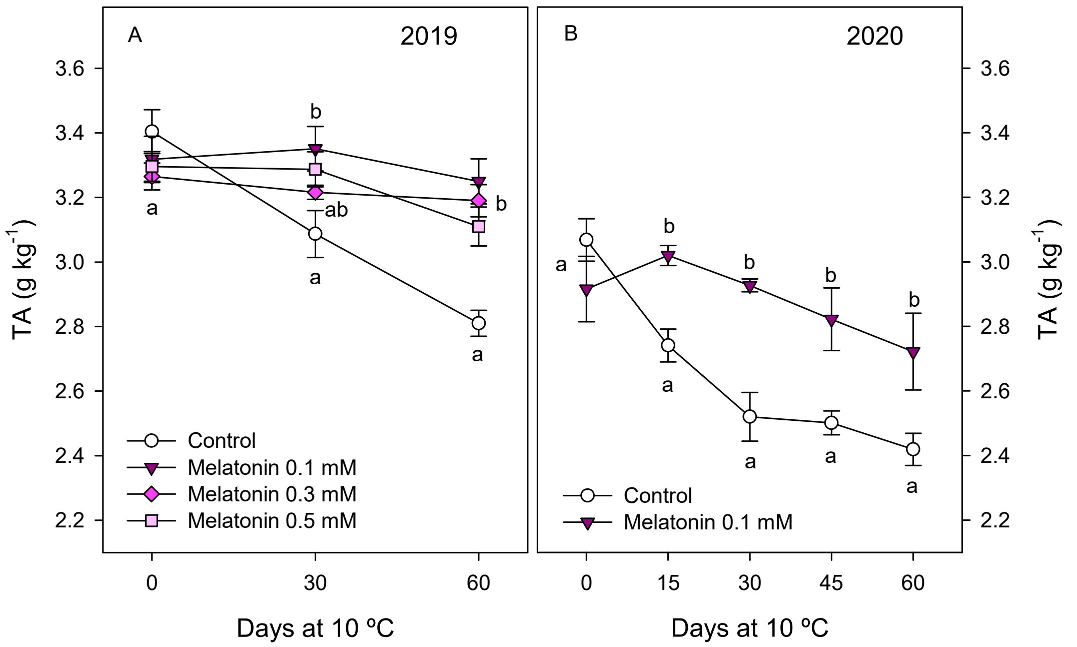

Figure S3. Titratable acidity (TA) in arils of pomegranate fruit from control and melatonin-treated trees during storage at 10 °C. Data are the mean ± SE. Different letters show significant differences (p < 0.05) among treatments for each sampling date.
